# Hsp90 as a master regulator in autoimmunity: from cellular stress to clinical opportunity

**DOI:** 10.1098/rsob.260040

**Published:** 2026-10-14

**Authors:** Scott Scarneo, Robert Freeze, Timothy A. Haystead

**Affiliations:** ^1^ Department of Research and Development, Eydis Bio Inc, Durham, NC, USA; ^2^ Department of Pharmacology and Cancer Biology, Duke University School of Medicine, Durham, NC, USA

**Keywords:** Hsp90, auto immune, inflammation, inhibitors

## Abstract

Heat shock protein 90 (Hsp90) is a ubiquitous molecular chaperone that governs the folding, stabilization and activity of a wide range of client proteins, many of which are critical regulators of immune cell signalling and inflammatory responses. Recent research has underscored the role of Hsp90 in orchestrating both innate and adaptive immune functions, particularly in the context of autoimmune diseases. In autoimmune diseases, dysregulated immune pathways often involve Hsp90-dependent client proteins, including key kinases and transcription factors. By modulating the activation, differentiation and survival of immune cells Hsp90 emerges as a central node in autoimmune pathogenesis. Importantly, the pharmacological inhibition of Hsp90 has shown promise in preclinical models of autoimmunity, where it attenuates pro-inflammatory pathways. In this review, we examine the immunoregulatory functions of Hsp90 with a focus on autoimmune disorders, highlighting its role in disease-driving pathways, such as Nuclear Factor Kappa B (NF-κB), Janus Kinase/Signal Transducer and Activator of Transcription (JAK/STAT) and Mitogen-Activated Protein Kinase (MAPK). We also discuss the current landscape of Hsp90 inhibitors, their mechanism of action, and emerging data on their efficacy in autoimmune disease models. Understanding how Hsp90 shapes immune cell behaviour and inflammation provides a compelling framework for its development as a novel therapeutic target in chronic autoimmune conditions.

## 1. Introduction

Heat shock protein 90 (Hsp90) is a highly conserved, ubiquitously expressed molecular chaperone that constitutes 1%–2% of the total cellular proteome under basal conditions and can increase under stressed conditions up to approximately 4%–6% [1–3]. Hsp90 is central to proteostasis, stabilizing and refolding nascent, misfolded and stress-damaged proteins. Structurally, Hsp90 is composed of an N-terminal adenosine triphosphate (ATP)-binding domain, a middle domain that facilitates client interaction and a C-terminal dimerization and substrate-binding domain [4,5]. The chaperoning function of Hsp90 is ATP-dependent, with ATP hydrolysis driving conformational changes that regulate client protein binding and release [6]. These activities are further modulated by co-chaperones, such as HOP, CDC37 and Hsp70, which confer substrate specificity and functional regulation [7–9]. Traditionally, Hsp90 has been studied in the context of protein kinases, transcription factors and other signalling proteins critical for cellular growth and survival. Recent discoveries, however, have highlighted its central role in immune cell biology, implicating Hsp90 as a critical regulator of both innate and adaptive immune responses [10,11].

Despite its ubiquitous expression, targeting Hsp90 has shown remarkable selectivity and tolerability *in vivo* [12,13]. This phenomenon may be attributed to the differential regulation of Hsp90 in stressed versus homeostatic cells [14]. In immune cells undergoing activation, the increased demand for protein synthesis and signalling creates a heightened dependency on Hsp90 chaperoning activity, rendering these cells more susceptible to Hsp90 inhibition. Furthermore, Hsp90 inhibitors exploit the proteostatic imbalance characteristic of inflamed tissues [15]. By destabilizing key signalling proteins, these inhibitors may selectively dampen hyperactive immune responses without broadly suppressing immune function. This property may be advantageous for treating autoimmune diseases, where targeted suppression of discrete immune responses is desirable. To date numerous Hsp90 inhibitors have been developed for the treatment of various cancers. Clinical data from these inhibitors shed light on the tolerability and safety of long-term Hsp90 inhibition. Here, we explore the current state of Hsp90 inhibitors used in the clinical setting as well as explore the role of Hsp90 as a novel target for the treatment of immune-mediated autoimmune diseases.

## 2. Hsp90 inhibition in humans is well tolerated despite ubiquitous expression

Hsp90 inhibitors have undergone extensive evaluation in human clinical trials, primarily in oncology, where they have been investigated for their ability to destabilize oncogenic proteins and promote cell death [13,16–18]. More than 23 distinct Hsp90 inhibitors, including geldanamycin derivatives such as 17-AAG (tanespimycin) and 17-DMAG, as well as synthetic small molecules like ganetespib, SNX-5422 and AUY922, have been tested in over 187 clinical trials across phases I–III [13,17,19]. These trials have collectively provided robust data on the safety, tolerability and pharmacokinetic profiles of these inhibitors in diverse patient populations.

Despite the ubiquitous expression of Hsp90 across all cell types, Hsp90 inhibitors have demonstrated a remarkably favourable safety profile in humans [17]. Several mechanisms have been proposed to explain the apparent therapeutic window of Hsp90 inhibitors despite ubiquitous expression. Activated immune cells and diseased tissues exhibit heightened proteostatic stress and greater dependence on Hsp90-supported signalling networks. This high-affinity multichaperone-complex state was originally demonstrated for tumour-derived Hsp90, which showed increased association with co-chaperones and markedly greater inhibitor affinity than Hsp90 from normal tissues [15]. Consequently, inhibition disproportionately affects pathogenic signalling pathways while relatively sparing quiescent cells.

Indeed, over 23 distinct Hsp90 inhibitors have entered clinical trials worldwide, derived from 5 distinct scaffolds. Recently, one Hsp90 inhibitor has been approved for gastrointestinal stromal tumours (GIST) in Japan [20]. The most common adverse effects reported in trials are mild and manageable, including temporary night blindness, fatigue, diarrhoea and transient elevations in liver enzymes [17,21–23]. Importantly, toxicity varies substantially by inhibitor scaffold and tissue exposure, making compound selection particularly relevant for chronic indications. Second-generation synthetic N-terminal inhibitors reduced the hepatotoxicity associated with geldanamycin-class compounds, in part through elimination of the quinone-containing geldanamycin scaffold, although hepatic adverse events have still been reported with individual agents [24]. Ocular toxicity has also emerged with several inhibitors, most notably AUY922, for which reversible night blindness, photopsia and blurred vision have resulted in dose modification, discontinuation or dose-limiting toxicity in some studies [25]. Preclinical comparisons suggest that retinal toxicity is associated with prolonged retinal drug exposure and slow elimination, whereas compounds with more rapid retinal clearance exhibit reduced photoreceptor toxicity [26]. Despite these compound-specific liabilities, severe haematological toxicity and sustained immunosuppression have not emerged as predominant class-wide toxicities. Collectively, these findings support the potential of Hsp90 inhibition in autoimmune and inflammatory diseases while emphasizing that scaffold selection, tissue pharmacokinetics and long-term safety will be critical considerations for chronic therapy.

The safety of Hsp90 inhibitors in oncology patients has been particularly noteworthy given the heightened physiological stress and comorbidities often present in this population. For instance, clinical trials of ganetespib in patients with advanced solid tumours and haematological malignancies demonstrated prolonged stable disease in responsive individuals, with tolerability allowing for continuous administration over months to years [27–29]. To date, the majority of Hsp90 clinical evaluation has been in oncology, however a phase 2 trial was completed in patients with hidradenitis suppurativa showing potential efficacy in improving disease status (table 1) [30]. Additionally, a phase 1b was completed with RGRN-305 in psoriasis that demonstrated reduced Psoriasis Area and Severity Index (PASI) scores in a subset of patients [32,36]. Other studies are currently in the preclinical stage. An example is the SNX series of inhibitors which was tested in the collagen-induced arthritis (CIA) and adjuvant-induced arthritis (AIA) rat models. In these studies, the group showed that Q2D treatment with SNX-4414 showed a dose-dependent reduction in disease activity with more than 90% inhibition of arthritis when dosed at 12 mg kg^−1^ p.o [34].

**Table 1.** Preclinical and clinical testing of Hsp90 inhibitors in immune-mediated disorders.

Hsp90 inhibitor	development status	route and dose	application in autoimmune or immune-mediated diseases	refs.
**ganetespib**	clinical trials completed	IV infusion 7–259 mg m^−3^ QW	evaluated in combination with chemotherapy for cancer; preclinical studies suggest potential in enhancing T-cell-mediated anti-tumour responses.	[30]
**RGRN-305**	clinical trial completed	oral, 250 mg QD for 16 weeks	phase 2 trial for hidradenitis suppurativa; results show HiSCR-50: 60% RGRN-305 versus 20% placebo.	[31]
**RGRN-305**	clinical trial completed	oral, 250 or 500 mg QD for 12 weeks	phase 1b for plaque psoriasis. 6 out of 11 showed PASI improvements ranging from 71% to 94%.	[32]
**EC144**	preclinical studies	—	synthetic inhibitor; shown to block innate immune responses in preclinical models.	[33]
**SNX-4414 & 7081**	preclinical studies	—	Hsp90 inhibition blocked CIA arthritis in a rat model of rheumatoid arthritis (RA), reduced levels of TNF in sera of animals	[34]
**HS-131**	preclinical studies	—	HS-131 blocked inflammatory pain in a complete Freund's adjuvant preclinical model and CIA arthritis	[14,35]

The safety and tolerability of Hsp90 inhibitors in oncology trials suggest that these agents could be repurposed for autoimmune and inflammatory diseases, where the therapeutic goal shifts from tumour cytotoxicity to immune modulation. Autoimmune diseases such as RA, systemic lupus erythematosus (SLE) and multiple sclerosis often involve chronic activation of immune signalling pathways, including NF-κB, JAK/STAT and MAPK, all of which are regulated by Hsp90 [37–39]. By selectively destabilizing these pathways in overactive immune cells, Hsp90 inhibitors may mitigate inflammation and autoimmunity while preserving normal immune function.

Another advantage of Hsp90 inhibitors in autoimmune diseases is their potential for tissue specificity. In inflamed tissues, such as arthritic joints or inflamed intestinal mucosa, immune cells are under significant proteostatic stress, creating a heightened dependency on Hsp90 chaperoning activity [40,41]. This stress-dependent selectivity could enable Hsp90 inhibitors to preferentially target hyperactive immune cells in diseased tissues, minimizing systemic effects. Moreover, their demonstrated tolerability in oncology trials suggests that Hsp90 inhibitors could be safely administered over extended periods, a crucial consideration for managing chronic autoimmune conditions [17,42,43]. Collectively, the clinical experience with Hsp90 inhibitors in oncology provides a strong foundation for their development in autoimmune and inflammatory diseases. Ongoing research into the immunomodulatory roles of Hsp90 and its client proteins will further refine our understanding of how these agents can be effectively repurposed for non-oncologic therapeutic applications.

## 3. Hsp90 client proteins in innate immune cell activation

Innate immune cells play a central role in the initiation and perpetuation of autoimmune diseases, where their activation and polarization towards pro-inflammatory phenotypes contribute to chronic tissue damage and disease progression [44]. Targeting the signalling pathways that govern their activation offers a promising strategy to dampen inflammatory responses and restore immune homeostasis. Hsp90 is a key regulator of this process, serving as a molecular chaperone that stabilizes and maintains the function of numerous kinases and transcription factors essential for innate immune signalling (table 2). A primary example is TAK1 (MAP3K7), a pivotal kinase activated downstream of toll-like receptors (TLRs) and interleukin receptors-1 (IL-1Rs). TAK1 is responsible for transmitting signals that activate both NF-κB and MAPK pathways, culminating in the production of pro-inflammatory cytokines in macrophages [38,39,69,70]. Hsp90 directly associates with TAK1, stabilizing its conformation and protecting it from proteasomal degradation, thus maintaining the cell's capacity to respond to pathogenic stimuli [45,71]. Similarly, upstream TLR effectors such as Interleukin-1 Receptor-Associated Kinase 1 (IRAK1), Interleukin-1 Receptor-Associated Kinase 2 (IRAK2) and Interleukin-1 Receptor-Associated Kinase 1 (IRAK3) also depend on Hsp90 for post-translational stability [46]. Inhibition of Hsp90 leads to degradation of these kinases, attenuating the signal transduction cascade and blunting NF-κB activation.

**Table 2.** Clients of innate immune cell activation.

kinases and signalling molecules		refs.
protein	role	
TAK1 (MAP3K7)	central in TLR/IL-1R signalling, activates NF-κB and MAPKs in macrophages.	[45]
IRAK1/IRAK2	key downstream kinases in TLR signalling.	[46]
IKKα, IKKβ, IKKε	components of NF-κB activation complex.	[47]
JNK (MAPK8/9/10)	activated downstream of TLRs, involved in pro-inflammatory cytokine production.	[48]
p38 (MAPK14)	critical for M1 polarization, cytokine production.	[49]
STAT1/STAT3/STAT5	important in both M1 (STAT1) and M2 (STAT3/5) responses.	[50,51]
mTOR	regulates macrophage metabolism and activation.	[52]
AKT (PKB)	especially AKT1 (M2-promoting) and AKT2 (M1-promoting).	[53]
ERK5	MAPK involved in survival and cytokine expression.	[54]
SYK	involved in Fc receptor signalling and inflammasome activation.	[55]
TBK1	involved in IRF3 activation and type I IFN production (important in antiviral macrophage responses).	[56,57]
LKB1 (STK11)	modulates macrophage metabolism and polarization.	[58]
AMPKα	generally anti-inflammatory; activation promotes M2-like phenotype.	[59]
PI3K/PDK1	upstream of AKT/mTOR, controls inflammatory signalling.	[52,60]
**transcription factors**		
NF-κB p65 (RelA)	master transcription factor for pro-inflammatory cytokines in M1 macrophages.	[61]
IRF2	IRF3 is involved in antiviral and inflammatory responses in macrophages.	[56]
HIF-1α/HIF-2α	hypoxia-driven responses; HIF-1α promotes glycolysis and M1 polarization, HIF-2α supports M2.	[62]
ATF3	negative regulator of TLR signalling; controls inflammation resolution.	[63]
FOXO1/FOXM1	FOX proteins in general are involved in metabolism and macrophage function	[64,65]
Nrf2	promotes antioxidant responses and M2 polarization.	[66,67]
PPARγ/PPARδ (nuclear receptors)	key in M2 macrophage polarization and lipid metabolism.	[68]

In parallel, the IKK complex—comprising IKKα, IKKβ, IKKγ (NEMO) and IKKε—represents another essential node in Hsp90-supported NF-κB signalling. Hsp90, often in complex with co-chaperone Cdc37, facilitates the maturation and stabilization of IKKβ, which in turn phosphorylates IκB, leading to its degradation and subsequent nuclear translocation of NF-κB subunits like p65 (RelA) [47,72,73]. This process is tightly regulated and essential for M1 macrophage activation. JNK (MAPK8/9/10) and p38 (MAPK14), other MAPKs activated downstream of TLRs, are similarly dependent on Hsp90 to maintain their phosphorylation-competent states [48,74]. In particular, p38 promotes M1 polarization and cytokine gene expression through transcriptional targets such as ATF2 and C/EBPβ [75].

Further downstream, Hsp90 also supports the activation and function of transcription factors that regulate gene expression programmes in activated macrophages. STAT1 is essential for the classical M1 response, particularly in response to IFN-γ, whereas STAT3 and STAT5 promote M2 polarization in response to IL-10 and IL-4 [76–79]. All three STATs are Hsp90 clients, and inhibition of Hsp90 impairs their phosphorylation and nuclear translocation [50,80,81]. Similarly, the master transcription factor NF-κB p65 (RelA), central to pro-inflammatory gene transcription, relies on the chaperoning activity of Hsp90 both directly and indirectly through stabilization of upstream kinases. Moreover, IRF3, which is critical for antiviral responses and type I interferon production via TBK1, forms a complex with Hsp90 to ensure its availability for rapid signal transduction [56].

Additionally, Hsp90 client proteins also regulate the metabolic and environmental adaptation of immune cells. For instance, AKT and mTOR—central regulators of cell growth, metabolism and macrophage polarization—are stabilized by Hsp90 to preserve signalling under inflammatory conditions [52,53,82]. AKT1 promotes anti-inflammatory M2 polarization, whereas AKT2 supports glycolytic, M1-like programmes [83]. Hsp90 inhibition destabilizes both, leading to defective polarization responses. Similarly, HIF-1α, which governs hypoxia-induced glycolysis and supports M1 macrophage functions, is stabilized by Hsp90 under stable and hypoxic conditions alike [84]. Without Hsp90, HIF-1α is rapidly degraded, curtailing pro-inflammatory metabolic adaptation. Through these multi-layered interactions, Hsp90 maintains not only signal transduction capacity but also the metabolic fitness of macrophages during immune activation.

## 4. Hsp90 clients involved in adaptive immune activation

Like Hsp90's role in the innate immune system, several key proteins critical to the adaptive immune system have been identified as clients of Hsp90 (table 3). For example, Hsp90 plays an indispensable role in T-cell receptor (TCR) signalling by stabilizing a suite of proximal kinases and downstream effectors that initiate and propagate adaptive immune responses [99,100]. LCK, a Src family kinase, is a pivotal initiator of TCR signalling in CD4⁺ and CD8⁺ T cells [101]. Upon antigen engagement, LCK phosphorylates the CD3 ITAMs, facilitating the recruitment and activation of ZAP-70, another essential tyrosine kinase. Both LCK and ZAP-70 are direct Hsp90 clients, and their functional integrity relies on Hsp90's chaperoning activity, which protects them from proteasomal degradation and ensures their correct folding and localization [85–87]. Inhibition of Hsp90 destabilizes this TCR signalosome, leading to impaired downstream activation, including reduced NF-κB, MAPK and Ca²⁺ signalling pathways, which are essential for T cell proliferation and cytokine production.

**Table 3.** Clients of adaptive immune cell activation.

kinases and signalling molecules		refs.
protein	**role**	
LCK	key kinase in TCR signalling in T cells	[85,86]
ZAP-70	TCR-proximal tyrosine kinase in adaptive signalling	[87]
CDK4	controls T cell cycle progression	[88,89]
CDK6	supports T cell entry into cell cycle from G1 phase	[90]
BTK	essential for B cell receptor signalling and development	[91]
SYK	mediates signalling downstream of BCR in B cells	[92]
MAP3K14 (NIK)	central kinase in noncanonical NF-κB signalling that regulates lymphocyte development, survival and activation.	[93]
**transcription factors**		
c-Myc	drives proliferation and survival of activated T cells	[94]
ERα	essential for T cell development and cytokine responses	[95,96]
GR (glucocorticoid receptor)	Modulates anti-inflammatory signaling in lymphocytes	[97,98]

Once TCR signalling is initiated, additional Hsp90 clients amplify the signal and link it to transcriptional responses and cell cycle progression. CDK4 and CDK6, which govern the G1-to-S phase transition, are crucial in proliferating activated T cells [88,89,102]. Their kinase activities depend on proper assembly with cyclins and structural stabilization by Hsp90 and its co-chaperones [88,90]. Simultaneously, kinases such as JNK and p38 MAPK are activated downstream to promote expression of inflammatory cytokines and to facilitate Th1 differentiation, especially in CD4⁺ T helper subsets. These kinases require Hsp90 for maintaining a functional conformation and for mediating stress and cytokine-induced responses [103]. Notably, the MAP3K14 (NIK) kinase, central to non-canonical NF-κB signalling, plays a role in lymphoid organogenesis and in supporting late-phase B and T cell responses—its expression is similarly regulated by Hsp90-dependent stabilization [104].

In B cells, Hsp90 regulates several kinases involved in B-cell receptor (BCR) signalling and antigen responsiveness. Notably, Spleen tyrosine kinase (SYK), a proximal mediator of BCR signalling, has been identified as an Hsp90 client protein, and Hsp90 inhibition disrupts SYK activity and tonic BCR signalling. Together with Bruton's tyrosine kinase (BTK), SYK is a critical component of the signalling network downstream of the BCR [92]. These kinases not only initiate calcium flux and ERK activation but also prime B cells for differentiation and antibody production. Both SYK and BTK are validated Hsp90 clients, and their inhibition via Hsp90 antagonists leads to disrupted BCR signal propagation and impaired B cell survival [92,105].

Finally, transcriptional regulation in adaptive immunity is intricately tied to Hsp90-supported nuclear factors. NF-κB p65 (RelA) is central to the induction of inflammatory genes in both T and B cells and is dependent on Hsp90 for cytoplasmic stabilization and nuclear translocation following IKK activation [106]. IRF3 and IRF2, which regulate type I interferon responses, also depend on Hsp90 for proper conformation and responsiveness to viral cues, particularly in antiviral CD8⁺ T cell responses [56]. PPARγ, although classically associated with metabolism, plays an increasingly appreciated role in regulatory T cell (Treg) function by repressing inflammatory gene transcription and promoting lipid metabolism essential for suppressive activity. Nuclear receptors such as ERα and GR are also Hsp90 clients and contribute to T cell development and resolution of immune responses via steroid hormone signalling [107,108]. Altogether, Hsp90 acts as a central gatekeeper across T and B lymphocyte compartments, ensuring that kinase cascades and transcriptional responses proceed efficiently and in coordination with extracellular cues.

## 5. Hsp90 regulation of key cytokines targeted in autoimmune diseases

Inflammatory cytokines such as TNF-α, IL-1β, IL-6, IL-8 and IL-17 are central to the pathogenesis of autoimmune diseases, and several biologics that target these cytokines have revolutionized treatment. For instance, TNF-α inhibitors (e.g. *etanercept, adalimumab* and *infliximab*) are FDA-approved for conditions like rheumatoid arthritis, psoriasis, inflammatory bowel disease and ankylosing spondylitis [109]. IL-6 blockade with *tocilizumab* is an established strategy in RA and juvenile idiopathic arthritis, while IL-1β inhibitors such as *anakinra* and *canakinumab* are used in autoinflammatory syndromes and selected autoimmune conditions [110,111].

These cytokines are not only downstream effectors of immune dysregulation, but are functionally dependent on intracellular signalling cascades maintained by the molecular chaperone function of Hsp90. Hsp90 plays a pivotal role in stabilizing and activating numerous client proteins that mediate these inflammatory pathways, including components of NF-κB (e.g. IKK complex), JAK/STAT and MAPK signalling. For example, Hsp90 ensures the stability of IKK, which is essential for NF-κB-driven transcription of TNF-α and IL-6 [72]. Similarly, Hsp90 modulates STAT3, a key driver of IL-6–dependent inflammation and stabilizes kinases such as p38 MAPK that regulate IL-1β and IL-8 production [112]. Inhibiting Hsp90 has been shown to disrupt these pro-inflammatory signalling networks**,** resulting in decreased cytokine production and immune cell activation in preclinical models of autoimmunity [34,113]. This suggests that Hsp90 inhibition could serve as a novel upstream therapeutic strategy—one that targets the signalling architecture driving multiple cytokines simultaneously, rather than neutralizing them individually.

### 5.1. Interleukin-8 (IL-8)

Hsp90 stabilizes key components of the MAPK and phosphatidylinositol 3-kinase/AKT (PI3K/AKT) signalling pathways, both of which are upstream regulators of IL-8 production [53]. Inhibition of Hsp90 has been shown to suppress IL-8 expression by disrupting these pathways. For instance, in head and neck squamous cell carcinoma cells, Hsp90 inhibition led to a blockade of glycolytic flux, which in turn reduced IL-8 levels and enhanced the infiltration of cytotoxic T cells, thereby potentiating the antitumour immune response [114].

Moreover, Hsp90 has been implicated in the regulation of IL-8 expression in response to environmental stimuli. Exposure to nickel ions (Ni²⁺) was found to increase IL-8 production through the binding of Ni²⁺ to Hsp90β, which enhances hypoxia-inducible factor 1-alpha (HIF-1α)-mediated IL-8 expression. This suggests that Hsp90β is a potential target for modulating Ni²⁺-induced inflammatory responses [115]. Additionally, in autoimmune conditions like bullous pemphigoid, Hsp90 has been identified as a crucial regulator of IL-8 production in keratinocytes. Blocking Hsp90 activity led to a significant decrease in IL-8 levels, suggesting that Hsp90 inhibitors could serve as potential therapeutic agents for treating various inflammatory skin diseases [116].

### 5.2. Interleukin-1 beta (IL-1β)

Hsp90 facilitates the proper folding and function of various client proteins involved in the IL-1β signalling cascade. Notably, Hsp90 inhibition has been shown to attenuate inflammation by modulating IL-1β pathways. For instance, in models of atopic dermatitis, the Hsp90 inhibitor RGRN-305 significantly suppressed the expression of pro-inflammatory cytokines, including IL-1β, leading to reduced inflammatory responses [117]. Furthermore, Hsp90 is implicated in the unconventional secretion of IL-1β. Studies have demonstrated that Hsp90, in complex with Cdc37, interacts with gasdermin D and the E3 ubiquitin ligase NEDD4 to facilitate the packaging of IL-1β into small extracellular vesicles for secretion [118]. This mechanism underscores the role of Hsp90 in the non-lytic release of IL-1β, contributing to the modulation of inflammatory responses [118].

### 5.3. Tumour necrosis factor (TNF)

As a molecular chaperone, Hsp90 ensures the stability and functionality of key components within the TNF signalling cascade. It is particularly important in stabilizing the IKK complex, which is required for NF-κB activation [73]. Through interactions with its co-chaperone Cdc37, Hsp90 stabilizes IKKα, IKKβ and the regulatory subunit NEMO, ensuring proper phosphorylation and degradation of IκB, a necessary step for NF-κB-mediated transcription of pro-inflammatory cytokines like TNF-α and IL-6 [72]. Hsp90 inhibitors, such as geldanamycin, have been shown to disrupt this process, impairing NF-κB activation and reducing cytokine production [73]. Hsp90 is also integral to the stabilization of receptor-interacting protein kinase 1 (RIPK1), a key mediator in TNF receptor 1 (TNFR1) signalling. RIPK1 governs the balance between pro-survival and pro-apoptotic signalling pathways. Hsp90 prevents RIPK1 degradation, ensuring effective downstream signalling to NF-κB [119].

The regulatory role of Hsp90 in TNF signalling has significant pathological and therapeutic implications. For instance, in RA, Hsp90 promotes osteoclast differentiation through the TNF receptor-associated factor 6 (TRAF6)/nuclear factor of activated T cells 1 pathway, exacerbating bone resorption and joint damage [120]. These findings highlight Hsp90 as a key modulator of TNF-driven inflammation and cell fate, suggesting its potential as a therapeutic target for chronic inflammatory diseases and autoimmune disorders characterized by dysregulated TNF signalling.

## 6. Hsp90's therapeutic potential in autoimmune diseases

Response to pathogens is a necessary biological response. However, in many diseases this protective cellular response may become dysregulated engendering a hyperactive pro-inflammatory state. Autoimmune diseases such as RA, ankylosing spondylitis, inflammatory bowel disease (IBD), psoriasis etc. have been shown to be mediated by elevated pro-inflammatory cytokine production. In particular, TNF has been targeted in the treatment of many of these diseases [121]. Treatments targeting TNF include *etanercept*, *adalimumab* and *infliximab* [122]. These biological-based therapies have been shown to dramatically reduce disease burden in responsive patients through inhibition of TNF signalling. However, many issues still plague anti-TNF biologics leading to upwards of 40% of patients either failing to respond to initial treatment or losing response over time owing to the development of anti-drug antibodies and other causes [123]. Thus, novel small molecule inhibitors of TNF and the signalling of other pro-inflammatory cytokines offer great potential in treatment of many autoimmune diseases. Preclinical studies have demonstrated that SNX-4144, a small-molecule Hsp90 inhibitor, significantly reduced disease burden in a rat model of RA by lowering pro-inflammatory cytokine levels, attenuating joint oedema and tissue damage and promoting recovery as evidenced by increased body weight [34].

Importantly, direct autoimmune-model evidence extends beyond the oncology literature. In myelin oligodendrocyte glycoprotein-induced experimental autoimmune encephalomyelitis, 17-AAG prevented disease onset when given early and reduced established neurologic disease [124]. In MRL/lpr lupus mice, 17-DMAG reduced proteinuria and anti-dsDNA autoantibodies, whereas ganetespib suppressed autoantibody production, lymphoid activation and renal injury in both prophylactic and therapeutic dosing paradigms [125,126]. EC144 similarly suppressed collagen-induced arthritis in mice, with reduced antigen-specific CD4+ T-cell activation and antibody responses, and Hsp90 inhibition ameliorated murine intestinal inflammation [34].

Furthermore, an eHsp90 targeting ligand, HS-131, showed upregulated eHsp90 expression on activated T cells as well as within the joints of CIA mice [14]. These findings suggest that expression of Hsp90 changes following immune cell activation with membrane ectopic expressed Hsp90 seen dramatically upregulated following T cell activation [14]. Furthermore, inhibition of eHsp90 with the selective ligand HS-131 showed a reduction in CIA disease burden. These studies support that conditional expression of Hsp90 under disease or stressed states may be preferentially targeted with tailored ligands such as HS-131.

Hsp90 inhibitors may represent a canonical means to target pro-inflammatory cytokine production. Previous Hsp90 clinical trials aimed at treating cancer have shown a high degree of tolerability to Hsp90 inhibitors coupled with relatively benign effects in healthy controls. Although at the time of writing, no Hsp90 inhibitors have been approved for autoimmune diseases, but mounting data implicating the role of Hsp90 in cytokine modulation provides a strong foundation for continued clinical exploration (table 4).

**Table 4.** Hsp90's role in autoimmune diseases.

autoimmune disease	evidence of Hsp90 involvement	key outcomes	refs.
**rheumatoid arthritis**	Elevated levels of mHsp90 on T cells and synovial fibroblasts; stabilization of pro-inflammatory signalling proteins	Hsp90 inhibition reduces IL-6, TNF-α and IL-1β production; decreases T cell activation and joint inflammation	[14,34]
**systemic lupus erythematosus**	elevated Hsp90 expression in peripheral blood mononuclear cells	Hsp90 in SLE has been associated with elevated IL-6 levels and the presence of autoantibodies targeting Hsp90.	[127–130]
**inflammatory bowel disease**	Hsp90 stabilizes IKK and STAT3, which regulate inflammatory cytokines like IL-6 and TNF-α	inhibition of Hsp90 reduces intestinal inflammation and cytokine production in preclinical models of colitis	[131]
**psoriasis**	elevated Hsp90 levels in lesional skin and keratinocytes; activation of STAT3	Hsp90 inhibition reduces keratinocyte proliferation and inflammatory cytokines like IL-17 and IL-22	[117,132–134]
**Type 1 diabetes**	elevated Hsp90 expression in pancreatic islets and peripheral immune cells	Hsp90 inhibition protects against beta-cell destruction by reducing IL-1β and TNF-α production	[135,136]

## 7. Selective Hsp90 targeting

Given the broad client repertoire and diverse immunoregulatory functions of Hsp90, the therapeutic consequences of its inhibition are highly dependent on how the chaperone is targeted. Differences in inhibitor binding site, paralog selectivity and effects on the heat-shock response can produce distinct patterns of client destabilization and immune modulation. These considerations have driven interest in more selective approaches to Hsp90 targeting that may preserve therapeutic efficacy while limiting the consequences of global chaperone inhibition.

N-terminal Hsp90 inhibitors bind the conserved N-terminal ATP-binding pocket and disrupt the ATP-dependent chaperone cycle, resulting in destabilization and proteasomal degradation of susceptible client proteins. N-terminal inhibition also commonly activates HSF1 and induces a heat-shock response characterized by increased expression of Hsp70 and other stress-response proteins [137]. In autoimmune models, N-terminal inhibitors including 17-AAG and 17-DMAG have reduced inflammatory responses and disease severity [124,138]. In some of these studies, therapeutic effects were accompanied by Hsp70 induction, suggesting that activation of the heat-shock response may contribute to, rather than solely oppose, the anti-inflammatory effects of Hsp90 inhibition. However, the relative contributions of client-protein depletion and HSF1-dependent stress responses remain incompletely defined.

C-terminal Hsp90 inhibitors represent a mechanistically distinct class that interact with the C-terminal region and allosterically alter Hsp90 chaperone function [139]. The C-terminal domain participates in Hsp90 dimerization and co-chaperone interactions, although the precise binding sites and mechanisms of many C-terminal inhibitors remain incompletely characterized [140,141]. Some C-terminal inhibitors have been reported to destabilize Hsp90 client proteins with less induction of the canonical heat-shock response than N-terminal inhibitors; however, this property is compound-dependent, and other C-terminal modulators can induce Hsp70 [140]. C-terminal inhibitors therefore provide an alternative approach to modulating Hsp90 function, but their therapeutic relevance to autoimmune disease remains largely unexplored and requires direct comparison with N-terminal inhibitors in autoimmune models.

Emerging evidence has suggested that individual Hsp90 paralogs regulate distinct immune functions through differential client repertoires and subcellular localization. For example, the ER-resident Hsp90 paralog GRP94 (gp96, HSP90B1) has a well-defined immune-specific client repertoire. Genetic studies have shown that GRP94 (gp96, HSP90B1) is required for the folding and surface expression of multiple Toll-like receptors and integrins, thereby regulating innate immune sensing, leukocyte adhesion and immune-cell development [142]. In gp96-deficient haematopoietic cells, defects in TLR and integrin expression alter immune-cell function, supporting the concept that targeting a specific Hsp90 paralog can selectively affect defined immune pathways rather than globally suppressing proteostasis [143].

Similarly, Hsp90β-selective inhibition has been reported to produce immune effects distinct from pan-Hsp90 inhibition In tumour models, the Hsp90β-selective inhibitor NDNB1182 increased interferon-response gene expression and enhanced antitumour immune responses, suggesting that selective targeting of Hsp90β can modulate immune signalling without necessarily impairing protective immunity [144]. Further immune-profiling studies across autoimmune, inflammatory and cancer models will be critical to defining the differential therapeutic efficacy and immunologic consequences of targeting individual Hsp90 family members.

## 8. Conclusion

Hsp90 plays a critical role in the regulation of immune cell function and the signalling networks that mediate both innate and adaptive immunity. Its essential role as a molecular chaperone extends to the stabilization and activation of numerous proteins involved in inflammatory signalling pathways, including NF-κB, MAPK and JAK/STAT, as well as those required for cytokine production and immune cell activation (figure 1). These functions make Hsp90 a key modulator of immune responses, with implications for both immune cell-mediated diseases and the broader inflammatory milieu.

**Figure 1.**
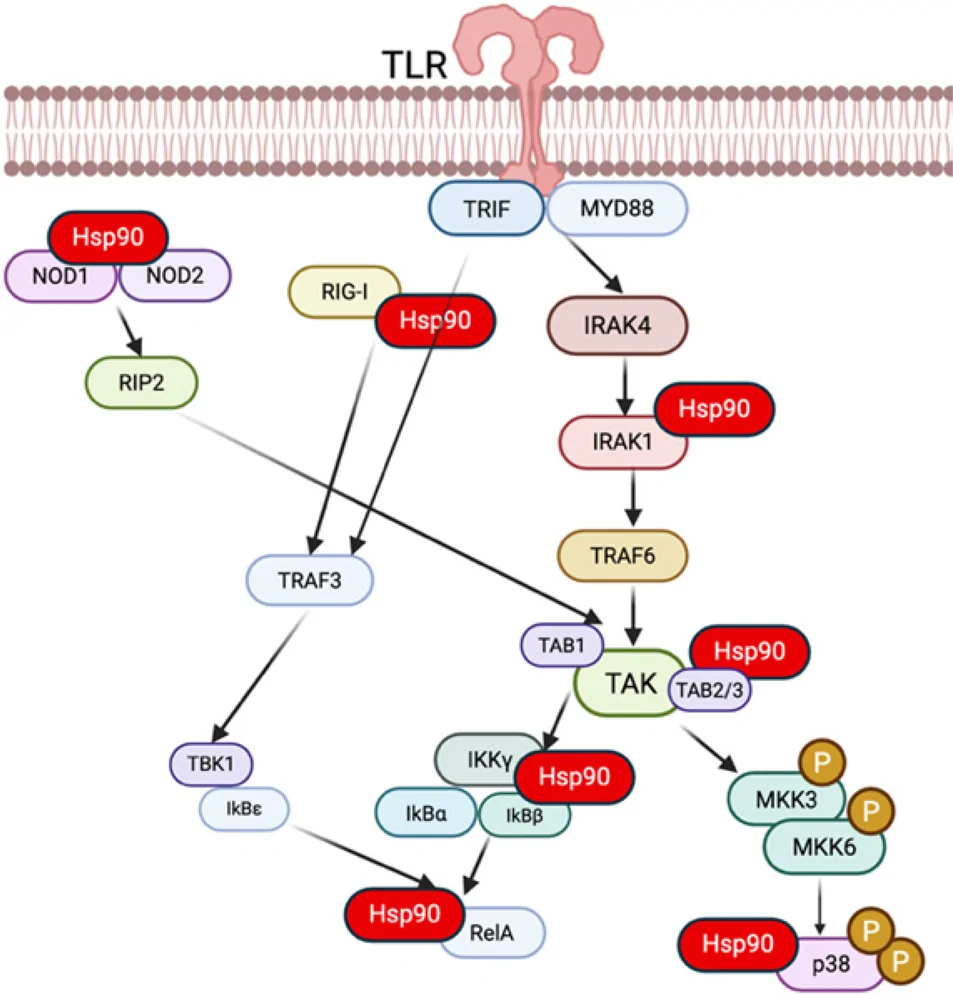
Hsp90 clients within the TLR signalling pathway. This figure illustrates the extensive involvement of the molecular chaperone Hsp90 in supporting the stability and function of key signalling proteins downstream of TLR activation. Hsp90 interacts with a wide range of client proteins across multiple nodes in the TLR cascade, including cytosolic pattern-recognition receptors (e.g. NOD1/2, RIG-I), kinases (e.g. p38, IRAK1/4, TAK1) and transcription factors (e.g. RelA).

The dysregulation of Hsp90 activity has been implicated in various immune-mediated diseases, including RA, systemic lupus erythematosus and IBD. Hsp90's role in supporting pro-inflammatory cytokine production, such as IL-6, TNF-α and IL-8, underpins its involvement in chronic inflammation and autoimmunity. Moreover, its emerging role in extracellular functions, including its expression on activated immune cells, provides a novel avenue for therapeutic targeting. eHsp90 on T cells and other immune cells could serve as a marker for activation and a potential target for modulating immune responses in diseases such as RA, where immune cell activation drives pathology.

Importantly, the therapeutic potential of Hsp90 inhibitors has already been demonstrated in oncology, with extensive clinical trials showing favourable safety and tolerability profiles. These findings provide a strong foundation for repurposing Hsp90 inhibitors for immune cell-mediated diseases. By targeting Hsp90, it may be possible to suppress pathological inflammation while preserving critical immune homeostasis. Additionally, the dual role of Hsp90 in regulating both pro-inflammatory and anti-inflammatory pathways highlights its potential as a modulator rather than a suppressor of immune responses.

Given the complexity of Hsp90's functions in immune cells and its involvement in diverse signalling pathways, future research is needed to delineate the context-dependent effects of Hsp90 inhibition. Studies focusing on immune-specific roles of Hsp90, such as its impact on cytokine production, immune cell trafficking and antigen presentation, will provide critical insights into its therapeutic utility. Overall, targeting Hsp90 in immune cells represents a promising strategy for treating a range of immune cell-mediated diseases, offering the potential to modulate disease progression and improve patient outcomes. Future development should therefore prioritize not only pan-Hsp90 inhibition but also paralog/isoform-selective approaches, including Hsp90β- or GRP94-selective inhibitors, and domain-selective strategies that distinguish N-terminal from C-terminal inhibition. Such approaches may preserve disease-relevant client destabilization while limiting ocular or hepatic toxicity, compensatory heat shock response (HSR) activation or disruption of homeostatic Hsp90 functions, and should be evaluated directly in autoimmune models.

## Data Availability

This article has no additional data.
